# Cognitive performance of post-covid patients in mild, moderate, and severe clinical situations

**DOI:** 10.1186/s40359-024-01740-7

**Published:** 2024-04-26

**Authors:** Antonio de Pádua Serafim, Fabiana Saffi, Amanda Rafaella A. Soares, Alessandra Mara Morita, Mariana Medeiros Assed, Sandro de Toledo, Cristiana C. A. Rocca, Ricardo S. S. Durães

**Affiliations:** 1 Institute of Psychology, University of São Paulo, São Paulo, Brazil; 2 Institute of Psychiatry, University of São Paulo, São Paulo, Brazil; 3 Institute of Psychology, University of Brasília, Brasília, Brazil

**Keywords:** COVID-19, Long COVID, Neuropsychological assessment, Cognitive difficulties

## Abstract

**Background:**

Studying individuals with varying symptoms, from mild to severe, can provide valuable insights into the spectrum of cognitive outcomes after COVID-19. We investigated the cognitive performance of adults who recovered from the novel coronavirus disease (COVID-19) without prior cognitive complaints, considering mild (not hospitalized), moderate (ward), and severe (intensive care unit) symptoms.

**Methods:**

This cross-sectional study included 302 patients who recovered from COVID-19 (mild, *n* = 102; moderate, *n* = 102; severe, *n* = 98). We assessed intellectual quotient (IQ), attention, memory, processing speed, visual-constructive ability, as well as symptoms of depression, anxiety, and stress, at least eighteen months after infection. The mean length of hospitalization was *M*_days_=8.2 (*SD* = 3.9) and *M*_days_=14.4 (*SD* = 8.2) in the moderate and severe groups, respectively.

**Results:**

Cognitive difficulties were present in all three groups: mild (*n* = 12, 11.7%), moderate (*n* = 40, 39.2%), and severe (*n* = 48, 48.9%). Using Multinomial Logistic Regression and considering the odds ratio, our results indicated that a one-point increase in sustained attention, visual memory, and working memory might decrease the odds of being categorized in the severe group by 20%, 24%, and 77%, respectively, compared to the mild group.

**Conclusions:**

Our findings provide empirical evidence regarding the long-term cognitive effects of COVID-19, particularly in individuals experiencing severe manifestations of the disease. We also highlighted the need for a comprehensive, multidimensional approach in rehabilitation programs to address the enduring cognitive impacts of COVID-19.

## Introduction

The cognitive impact after COVID-19 has become a subject of study and growing concern. Recent evidence has identified a set of characteristics, including long-lasting symptoms in individuals infected with the novel coronavirus disease Sars-CoV-2 (hereafter, COVID-19) [1–5], including cognitive impairment associated with recovered patients who required hospitalization [5–9]. Many individuals who recovered from the illness and needed hospitalization have reported persistent cognitive symptoms [6–10].

More than half of hospitalized patients continue to experience neurological symptoms for up to three months after the acute stage of COVID-19 [11]. Alemanno et al. [12] assessed cognitive function one month after discharge in patients with severe COVID-19 and found that 80% of these patients demonstrated cognitive deficits, particularly in memory, executive function, and language, using the Montreal Cognitive Assessment (MoCA) for cognitive screening.

A meta-analysis of 27 studies (resulting from the analysis of 6,202 articles) assessed a sample of 2,049 individuals, with a mean age of 56.05 years, examining their cognitive function up to seven months after infection [13]. The MoCA results demonstrated that people infected with COVID-19 had worse overall cognitive functioning than those who were not infected. The authors verified that higher age was associated with worse cognitive performance in these samples through a regression analysis. Additionally, in describing case series (*n* = 9) of COVID-19 using screening tests (Mini-Mental State Examination - MMSE), Negrini et al. [14] observed general cognitive decline in 33.3% of patients with pathological scores regarding attention, memory, language, and praxis skills. The deficit in general cognitive functioning was associated with the length of stay in the Intensive Care Unit (ICU). That is, the longer the length of stay in the ICU, the lower the MMSE score, indicating lower general cognitive performance.

Studies using neuropsychological batteries show that infected people suffer cognitive difficulties. A cross-sectional/longitudinal study investigated cognition in 130 women after COVID-19 infection (74% stated that they had “long COVID”) and 118 women with no history of COVID-19 aged 18–60 years for both groups. The COVID-19 group showed the worst results regarding reaction time and wordlist recognition memory [15].

Researchers in Argentina assessed the cognitive functions of 45 post-COVID patients 142 days after the illness and compared them to a control group of 45 participants and found that the COVID group performed worse on memory, attention, and executive function tests [16]. A Brazilian study, in which neuroimaging and cognitive tests were conducted on 61 COVID-19 patients with a mean of 59 days after diagnosis showed that 28% of participants had deficits in immediate episodic verbal memory, sustained and alternating attention, and cognitive flexibility [17]. Another study analyzed mental health and cognitive factors in 425 COVID-19 survivors between six and nine months after the acute phase of infection. Approximately 51.1% of participants reported subjective memory complaints [10].

In addition, several patients with mild COVID-19 symptoms without hospitalization have exhibited cognitive deficits. For example, in a study of 100 COVID-19 patients who did not require hospitalization, with a mean age of 43 years, Graham et al. [18] found that 53% had impairments in short-term memory and attention-related tasks. Hellmuth et al. [19] reported two cases with persistent cognitive complaints at 70 and 100 days after infection.

Based on current knowledge, the intricate interplay of various pathophysiological mechanisms associated with COVID-19 is linked to disruptions in brain homeostasis, resulting in cognitive symptoms and other manifestations. These mechanisms might establish connections with diverse pathways, leading to cognitive impairments, particularly in attention, working memory, and language-related attentional areas. Within this framework, factors such as the neuroinflammatory response triggered by the virus [1, 11, 20, 21]. For example, a study showed elevated chemokine levels in the cerebrospinal fluid, triggering microglial activation in the subcortical and hippocampal white matter regions [23]. According to the authors, microglial activation in the hippocampus might explain memory impairments in affected individuals. Complications of the disease that required ICU admission due to pneumonia-induced hypoxia [22–26], and the influential role of age [27, 28] play crucial roles.

Moreover, acute, and severe cases of SARS-CoV-2 infection may introduce psychiatric changes, encompassing depression, anxiety, stress, insomnia, and psychosis [10, 29–34]. This multifaceted spectrum of influences underscores the complexity of the impact of COVID-19 on cognitive function and mental health, highlighting the need for comprehensive research to unravel these intricate connections and pave the way for targeted interventions and support strategies.

Given the above, studying individuals with varying symptom severity, from mild to severe, can provide valuable insights into the spectrum of post-COVID-19 cognitive outcomes. This study aims to assess the cognitive performance of individuals affected by COVID-19, considering the different levels of disease severity, in a follow-up of at least 18 months after the infection, who did not have any previous cognitive complaints. Therefore, we present the following hypothesis: Individuals who experienced more severe forms of COVID-19 will exhibit a reduced cognitive performance compared to those who had mild to moderate forms 18 months after infection.

## Methods

### Sample size

The G*power software (version 3.1.9.7) was employed to calculate the statistical power of the study. We applied a = 0.05, medium effect size *f*^2^ = 18, 10 number of predictors, and aimed for a power = 0.80 for regression analyses. The estimated sample size for the current study was approximately *N* = 300.

### Participants

Cross-sectional data was collected from 302 volunteers (151 women and 151 men aged > 18 years) recruited from various social segments of public and private hospitals. The sample was categorized into three groups based on COVID-19 symptoms, according to the criteria established in Brazil throughout the pandemic: *mild* (*n* = 102), patients with fever, loss of smell and taste, runny nose, sore throat, head and muscle pain, abdominal issues, diarrhea, cough, or fatigue without requiring hospitalization; *moderate* (*n* = 102), patients experiencing persistent daily cough and fever with worsening breathing difficulties requiring medical care in the hospital; and *severe* (*n* = 98), patients who required admission to the ICU due to severe acute respiratory syndrome with O2 saturation below 94.

Eligible participants were aged 18 or older, approximately 18 months post-COVID-19 diagnosis confirmed by a positive RT-PCR test for SARS-CoV-2, along with medical documentation detailing the treatment administered during the infection. Exclusion criteria included cognitive or neuropsychiatric deficits predating the COVID-19 outbreak. All participants who agreed to participate in the study signed an informed consent form before the assessment.

### Measures

#### Demographic and clinical information

The battery of cognitive tests was organized based on data from the literature on attention, memory, and processing speed difficulties associated with COVID-19.

#### Intellectual quotient (IQ)

The Wechsler Abbreviated Intelligence Scale (WASI) [35] was used to assess the participants’ IQ, two subtests were used: (1) vocabulary, which assesses language development, semantic knowledge, and general intelligence (crystallized), and (2) matrix reasoning, assessing the visual perception of abstract stimuli (fluid intelligence). Higher scores indicate better performance.

#### Working memory

Digits Span - (DS) [36]. Wechsler Adult Intelligence Scale III is a scale to assess verbal working memory in forward and backward formats. The total Digit Span raw score (total number of items/list correctly repeated) was based on the sum of the Digits Forward and Digits Backward raw score. Higher scores indicate better performance.

#### Processing speed

*Coding Subtest* - *Wechsler Adult Intelligence Scale III.* [36]. It was used to assess processing speed, as well as the ability to follow instructions under time pressure, selective attention, concentration (resistance to distractibility), and motor persistence in a sequential task. Higher scores indicate better performance.

#### Sustained attention

The Sustained Attention Test (SA) assesses an individual’s ability to focus, select, and maintain attention on a specific target when facing simultaneous visual stimuli [37]. The test involves locating symbols presented among all symbols on the answer sheet based on three models presented over 2.30 min, standardized for the Brazilian population. Higher scores indicate better performance.

#### Visuo-constructive ability and visual-spatial memory

*Rey-Osterrieth Complex Figure* (ROCF) [38]. The ROCF is widely used to assess visuo-constructive ability (copy tests) and visual-spatial memory (recall tests, after 3 min of copying). The figure comprises 18 items that make up the whole figure. Items are scored from 0, 0.5, 1 or 2. Scores range from 0 to 36, reflecting accuracy and positioning of each item in the figure, with higher scores indicating better performance.

#### Depression, anxiety, and stress

*Depression, Anxiety, and Stress Scale − 21* (DASS-21) [39]. The DASS-21 assesses anxiety, depression, and stress. This scale contains 21 items divided into three subscales and uses a 4-point *Likert* scale. Each subscale consists of seven items that assess symptoms related to depression, anxiety, and stress. It provides three scores (one for each subscale), the total sum of which ranges between 0 and 21. Higher scores on each scale correspond to more negative or severe affective states.

### Procedures

Recruitment was conducted through social networks and hospital patient lists, with 326 respondents contacted via email or WhatsApp. A meeting via an online platform was arranged to explain the study’s details and verify inclusion criteria. After the first meeting, 16 individuals had not responded to various scheduling attempts. Thus, only 310 eligible individuals agreed to participate. For the next stage, we scheduled an initial in-person session to collect sociodemographic and clinical information, age, sex, marital status, educational level, occupation, history of previous neurological or psychiatric disease, type of COVID-19 symptoms, type of treatment (ward or ICU), length of stay (days in the ward or ICU), number of relatives infected with COVID-19, number of relatives who passed away due to COVID-19, data from imaging tests, cognitive complaints, psychiatric complaints, current medications, and analysis of medical reports. Of the 310 eligible individuals, five were excluded for not presenting evidence of a positive RT-PCR result for SARS-CoV2, and three did not return for their psychological symptoms and cognitive assessment, resulting in a final sample of 302 participants. For each participant, a neuropsychological battery assessed intellectual quotient (IQ), sustained attention, working memory, visual memory, visual-constructive ability, processing speed, and depression, anxiety, and stress symptoms.

### Statistical analysis

Data processing was conducted using IBM SPSS Statistics for Windows, version 23 (IBM Corp., Sao Paulo, S.P., BRA). Raw scores for each variable were employed for analysis after confirming normality through Shapiro-Wilk tests. Regarding to IQ and cognitive difficulties (see Tables 1 and 2), it has been used Brazilian standardized scores.

Multinomial Logistic Regression (MLR) was performed, after verifying assumptions, to explore interactions between independent variables in predicting the dependent variables [40]. Consider a logistic regression model to predict the probability of clinical situation based on cognitive, clinical, and sociodemographic factors. The dependent variable is multinominal (1 for mild, 2 for moderate, and 3 for severe), and the independent variables are continuous. The general formula interaction term could be: log(P)=*β*_0_+*β*_1_ × _1_+*β*_2_ × _2_+⋯+*β*_*p*_X_*p*_+*β*_*p*_ + 1(X_1_×X_2_)+⋯+*β*_*p*_+_*q*_(X_*p*_×X_*p*_+_1_) which *β*_*p*_+_1_, *β*_*p*_+_2_…, *β*_*p*_+_*q*_ are the interaction coefficients, and (X_1_×X_2_), (X_*p*_×X_*p*_+_1_), …, are the interaction terms between the variables. This term allows analyzing whether the effect of cognitive, clinical, and sociodemographic factors on the dependent variable varies according to clinical situation (mild, moderate, and severe) [41]. Effects of factors on the dependent variable were expressed as odds ratios (OR). A model was developed to explain the relationship between the independent and dependent variables. An OR of 1 signifies no effect on odds, OR > 1 denotes higher odds, and OR < 1 suggests lower odds. Regression coefficients estimated the increase in log odds with each unit increase in exposure. The exponential function of these coefficients provided the OR associated with a one-unit (one score point) increase in exposure [42].

After checking all assumptions, one-way multivariate analysis of covariance (MANCOVA) was used to determine whether there are any statistically significant differences between the adjusted means of three independent (unrelated) groups, while controlling for age. The effect size was assessed using Partial Eta square (η^2^), with interpretations as follows: >0.01 = small effect, > 0.06 = medium effect, and > 0.14 = large effect [43].

We employed three nominal categories for independent variables (mild, moderate, and severe), 10 dependent continuous variables (five cognitive, three psychological, and two sociodemographic). Multicollinearity was not observed in the data, while the significance level was set at *p* < 0.05.

## Results

### Demographic results and clinical aspects

Socio-demographic data, IQ measurements, and clinical information of the 302 participants who recovered from COVID-19 were assessed at least 18 months after infection (see Table 1).

**Table 1:** Sociodemographic data, IQ, and clinical severity by group (*N* = 302)

Variables		Mild_(not hospitalized)_	Moderate_(ward)_	Severe_(ICU)_
		(*N* = 102)	(*N* = 102)	(*N* = 98)
**Age**	Years old (*M*[*SD*])	46.7 (12.4)	50.1 (14.0)	55.3 (12.2)
**Educational level**	Years (*M*[*SD*])	14.4 (2.6)	12.4 (3.4)	11.6 (3.9)
**IQ** (*M*[*SD*])		96.7 (4.2)	95.3 (4.9)	95,4 (4.9)
**Clinical situation**	Not hospitalized (*n*[%])	102 (100)	–	–
	Ward - days (*M*[*SD*])	–	8.8 (3.6)	–
	ICU - days (*M*[*SD*])	–	–	15.2 (9.6)

IQ: intellectual quotient; ICU: intensive care unit; M: mean; SD: standard deviation

The participants were categorized according to disease severity, considering mild (not hospitalized), moderate (ward), and severe (intensive care unit) symptoms at least eighteen months after infection (Table 1). The sample comprised individuals, both male and female, with high educational levels and without cognitive complaints prior to contracting COVID-19. The total sample size was *N* = 302 (*n* = 151_men_, 50% and *n* = 151_women_, 50%). There were no IQ differences in the three groups.

### Cognitive assessment and psychological aspects

Table 2 illustrates the distribution of participants across each group, analyzing cognitive difficulties (a lower score based on standardized) according to scores below the normative mean values in Brazil for each instrument used.

**Table 2:** Description of cognitive difficulties and depressive symptoms among groups

Variables	Mild_(not hospitalized)_	Moderate_(ward)_	Severe_(ICU)_
	(*N* = 102)	(*N* = 102)	(*N* = 98)
	*n* (%)	*n* (%)	*n* (%)
General distribution^¶^	12 (11.9)	40 (39.6)	46 (48.4)
Cognitive processing speed_(Coding)_	2 (2.0)	9 (8.9)	17 (17.9)
Sustained attention	4 (3.9)	9 (8.9)	32 (33.7)
Visuo-spatial construction_(ROCF−C)_	5 (5.0)	19 (18.8)	30 (31.6)
Visual memory_(ROCF−R)_	0	3 (3.0)	17 (17.9)
Working memory_(DST)_	1 (1.0)	29 (28.7)	41 (43.2)
Depression_(DASS−21)_			
Mild symptoms	1 (1.9)	3 (3.0)	8 (8.4)
Moderate symptoms	0	7 (7.0)	12 (13.6)
Severe symptoms	0	7 (7.0)	10 (10.5)

ROCF-C: Rey Figure copy. ROCF-R: Rey Figure recall. DST: Digit Span total

^¶^: number of participants that presented cognitive difficulties at least in one variable or depressive symptom

The data from Table 2 indicate higher percentages of cognitive difficulties in the severe group, followed by the moderate group, compared to the mild group. Notably, even within the mild group, 11% of participants exhibited difficulties in at least one assessed cognitive function 18 months after COVID-19 infection. No clinical signs of severe anxiety and stress were observed across the three groups.

The Multinomial Logistic Regression model fitting information showed an a-2 log-likelihood = 374.773, c^2^ = 288.683, *df* = 20, *p* = 0.000, indicating an adequate predictor model. It was confirmed by the Pearson Goodness-of-Fit test (c^2^ = 489.798, *df* = 582, *p* = 0.998). Pseudo R^2^ estimates were calculated (Cox and Snell = 0.616, Nagelkerke = 0.693, and McFadden = 0.435). Thus, the variation in the groups might be explained by the full model, suggesting that the predictions were reliable. The model accurately predicted 73.5% of the cases in their respective groups. Specifically, 85.3% of the cases were correctly predicted as mild_(not hospitalized)_, while 57.8% and 77.6% of the cases were correctly predicted as moderate_(ward)_ and severe_(ICU)_, respectively.

**Table 3:** Likelihood Ratio Tests

Variables	Model Fitting Criteria	Likelihood Ratio Tests		
	-2 Log Likelihood of Reduced Model	c^2^	df	*p*
Processing speed	384.986	10.214	2	**< 0.01**
Sustained attention	418.962	44.190	2	**< 0.001**
Visuospatial construction_(ROCF−C)_	380.883	6.111	2	0.047
Visual memory_(ROCF−R)_	426.755	51.983	2	**< 0.001**
Working memory_(DST)_	400.562	25.789	2	**< 0.001**
Depressive symptoms	388.496	13.723	2	**< 0.001**
Anxiety symptoms	375.842	1.069	2	0.586
Stress symptoms	384.730	9.958	2	**< 0.01**
Age	391.537	16.764	2	**< 0.001**
Educational level_(years)_	380.284	5.511	2	0.064

The chi-square statistic is the difference in -2 log-likelihoods between the final model and a reduced model. A reduced model was formed by omitting the effects of the final model. The null hypothesis was that all parameters of this effect were zero

Table 3 presents the likelihood ratio tests conducted to assess the contribution of each variable effect to the model. For each one, a -2 log-likelihood was calculated for the reduced model, representing a model excluding the variable effect, that is, log-likelihood statistic was compared between model that either did or did not contain the variables to determine if the variables contributed significantly to the model. If the significance level of the test was < 0.05, the effect contributed to the model. Thus, processing speed, sustained attention, visual memory, working memory, depression, stress, and age, were observed to make comprehensive contributions to the model.

**Table 4:** MLR analysis for cognitive, psychological, and socio-demographic variables in mild_(not hospitalized)_, moderate_(ward)_, and severe_(ICU)_ groups of post COVID-19 patients

Group^a^	b	SE	*p*	OR	95% CI for OR	
					Lower	Upper
**Moderate**_**(ward)**_						
Processing speed	− 0.060	0.023	**< 0.01**	0.942	0.900	0.986
Sustained attention	− 0.075	0.028	**< 0.01**	0.928	0.877	0.981
Visuospatial construction_(ROCF−C)_	0.102	0.079	0.198	1.107	0.948	1.292
Visual memory_(ROCF−R)_	− 0.047	0.037	0.196	0.954	0.888	1.025
Working memory_(DST)_	− 0.203	0.075	**< 0.01**	0.817	0.705	0.946
Depressive symptoms	0.307	0.096	**< 0.001**	1.360	1.127	1.641
Anxiety symptoms	0.088	0.096	0.360	1.092	0.905	1.317
Stress symptoms	0.262	0.088	**< 0.01**	1.769	1.648	1.914
Age	0.105	0.028	**< 0.000**	1.900	1.852	1.951
Educational level_(years)_	− 0.142	0.063	0.060	0.868	0.766	0.983
**Severe**_**(ICU)**_						
Processing speed	− 0.004	0.027	0.879	0.996	0.945	1.049
Sustained attention	− 0.224	0.039	**< 0.000**	0.799	0.741	0.862
Visuospatial construction_(ROCF−C)_	− 0.059	0.082	0.475	0.943	0.802	1.108
Visual memory_(ROCF−R)_	− 0.272	0.046	**< 0.000**	0.762	0.695	0.834
Working memory_(DST)_	− 0.203	0.103	**< 0.05**	0.226	0.001	0.500
Depressive symptoms	0.264	0.102	**< 0.01**	1.302	1.065	1.590
Anxiety symptoms	0.048	0.106	0.651	1.049	0.852	1.293
Stress symptoms	− 0.218	0.105	0.058	0.804	0.654	0.988
Age	0.084	0.033	**< 0.01**	1.920	1.862	1.981
Educational level_(years)_	− 0.137	0.074	0.064	0.872	0.754	1.008

^a^ The reference category is mild (Not hospitalized)

OR: odds ratio; SE: standard error; 95% CI: confidence interval

ROCF-C: Rey-Osterrieth Complex Figure-copy; ROCF-R: Rey-Osterrieth Complex Figure-recall; DST: digit span total (forward and backward)

Table 4 shows the results of the MLR, which was used to predict the probabilities of different possible outcomes of a categorically distributed dependent variable, given a set of independent variables. This can explain the relationship between a nominal dependent variable and one or more independent variables. OR is a measure of how strongly an event is associated with exposure. Specifically, it quantifies the ratio between two sets of odds: the odds of an event occurring in an exposed group versus those in a non-exposed group. It aims to ascertain the likelihood of exposure leading to a specific event [44].

In reference to the mild (not hospitalized) group as the baseline, the MLR results (Table 4) indicate that a one-unit increase in processing speed, sustained attention, and working memory performance could potentially reduce the odds by 6%, 7%, and 18%, respectively, of being categorized in the ward group compared to the group that was not hospitalized. However, it does not mean that increasing cognitive performance protects against disease severity. When reading the odds results of this study, for example, in the above results, it might be inferred that patients in the ward group tend to have poorer cognitive performance than the not hospitalized group. High levels of depressive and stress symptoms suggested being categorized in ward group, in comparison to the group of individuals who were not hospitalized. The results demonstrate that for each incremental point in depression and stress scores, the odds ratio increased by 36% and 77%, respectively, for being categorized in the ward group, considering the non-hospitalized group as the reference. It also suggests that patients in the ward group tend to have higher depressive and stress symptoms than not hospitalized group. Age was also found to increase the odds by 90% of being categorized in the ward group. Similar associations were observed for age and depressive symptoms in the ICU group, with an increase in odds ratio by 92% for age and 30% for depression.

In addition to age and depressive symptoms explaining why some individuals were placed in the severe group, three cognitive variables also were predictors explained for this specific sample, showing a negative relationship: sustained attention, visual memory, working memory (see Table 4). This means that with each improved performance point, there could be a reduction in the odds ratio for being categorized in the ICU group by 20%, 24%, and 77%, respectively. These results point out patients in the ICU group tend to present poorer cognitive performance than not hospitalized group.

A One-Way Multivariate Analysis of Covariance (MANCOVA) revealed a statistically significant distinction between the clinical groups (mild, moderate, and severe) concerning the combined cognitive variables, after controlling for age, *F*(16, 582) = 17.697, *p* < 0.000, Wilks’ Λ = 0.453, partial η^2^ = 0.327, indicating a large effect size. Upon analyzing *p*-value associated with the covariate ‘age’ on each dependent variable, the result was *p* < 0.000 for all cognitive variables with partial η^2^ > 0.14 (large effect), but with no significance for the clinical variables (depression, anxiety, and stress). On the other hand, after controlling for depressive symptoms, results presented no statistically significant changes (*p* = 0.165, partial η^2^ > 0.14) between groups, regarding cognitive variables.

## Discussion

This study stems from an exploratory analysis that revealed original data on people’s cognitive effects after varying degrees of SARS-CoV-2 contamination (mild, moderate, and severe) and without a history of cognitive complaints.

We showed that cognitive symptoms persist in mild cases and are even more prevalent in individuals with severe manifestations. Furthermore, we confirmed our central hypothesis: people with severe forms of COVID-19 show diminished cognitive performance 18 months after infection compared to those with mild to moderate forms.

Although identifying cognitive difficulties in mild cases, as observed in this study, aligns with the existing literature, this paper presents specificities that set it apart from these investigations. For instance, a study comparing 50 COVID-19 individuals to 50 healthy controls using a computerized neuropsychological battery [18] reported deterioration in processing speed, attention, executive function, and working memory in 53% of clinical cases, persisting at least six weeks post-symptom onset. Though our study also detected attention and working memory issues (digit span), we did not observe problems in visual memory tasks using the Rey-Osterrieth Complex Figure. Additionally, we demonstrated a notably lower incidence rate of cognitive problems (12%). This discrepancy suggests that cognitive difficulties may diminish over time in milder cases. It’s noteworthy that Hammerle et al. [29] employed cognitive screening tests different from our protocol.

Regarding cognitive measures, it is noteworthy that performance in tasks involving processing speed, attention, working memory, and visuoconstructive ability varied across the three groups. The moderate group exhibited poorer performance than the mild group in these aspects, while the severe group demonstrated an even lower performance than the moderate group.

When contextualizing our findings within the existing literature, we observe that the most prevalent cognitive difficulties in the severe group align with other studies, associating disease severity with age [27, 28], regardless of variations in the neuropsychological protocols. Notably, our study stands out as the first to analyze three severity groups simultaneously, providing a more comprehensive case perspective, distinct from previous studies.

Regarding cognitive aspects, performance in processing speed and working memory was similar between the moderate and severe groups in this study, both differing from the mild group. These findings suggest that, although all groups were affected by COVID-19, the effects on cognitive function may vary in intensity. However, it is crucial to highlight that the severity of symptoms and the post-COVID-19 cognitive impact can be influenced by various factors, such as age, hypoxia, and depressive symptoms [17, 18, 22, 25, 27, 29–32, 45–47]. While cognitive difficulties can vary from person to person, and other factors may influence these results, it is suggested that COVID-19 significantly contributes to mental challenges associated with long-term symptoms [2, 7, 8, 10, 12, 15, 16, 18, 19, 26, 34, 48].

Another factor we observed in our study, in line with the current literature, is the incidence of depressive symptoms, particularly among COVID-19 hospitalized patients [30–34]. We highlight that these symptoms were more prevalent in the severe cases group compared to moderate and mild cases. Overall, the COVID-19 pandemic significantly elevated stress and anxiety levels in both the general population and in patients with more severe symptoms requiring hospitalization [26, 28, 36, 45–49]. Depressive symptoms also might affect cognitive functioning, including attention, memory, processing speed, and decision-making [50]. However, in our study, even after controlling the depressive symptoms variable, there were no statistically significant changes in the cognitive variables. These results corroborate Cysique et al. [51] and Woo et al. [52] studies which showed cognitive impairments may manifest itself independently of depression after COVID-19 infection. Nevertheless, it is essential to consider mental health in conjunction with cognitive aspects when assessing the post-COVID-19 effects in both moderate and severe cases, as mentioned in the literature [30–34, 53, 54].

Better performance in processing speed, sustained attention, and working memory was associated to decreased odds of being categorized in the ward group compared to the mild group. Similarly, higher levels of depressive and stress symptoms were indicative of a higher likelihood of being categorized in the ward group. Exploring cognitive predictors within the severe group, our investigation identified sustained attention, visual memory, and working memory as pivotal factors, demonstrating a negative association with the ICU group. Improvements in these cognitive domains were correlated with a substantial reduction in the odds of being categorized in the ICU group.

We observed a significant association between depressive symptoms, age, and hospitalization groups. An increase in these symptoms translated into more than a third of a chance of belonging to the ward group. Our observations indicated a gradient of cognitive difficulties and depressive symptoms with lower occurrences in the mild group, increased occurrences in moderate cases, and a higher incidence in severe cases, as post-COVID consequences [18, 55, 56]. Furthermore, we emphasize that the presence of cognitive symptoms and other conditions are not only associated with severe cases of COVID-19 [5, 32].

In addition to depressive symptoms, age was also an important variable. For example, each additional year of age was associated with a greater probability of belonging to the ward group, with the non-hospitalised group as the reference point. In the ICU group, both age and depressive symptoms exhibited similar patterns. Older Individuals were more likely to experience the severe form and more cognitive difficulties. A study review reported that adults over 65 represent 80% of hospitalisations and have a 23 times greater risk of death than younger people [27]. A post-hospitalization by COVID-19 study (between 5 and 12 months) with 2,320 individuals (mean age 58.7 ± 12.5 years) demonstrated that the mean age was higher (67.8 ± 11.4 years) among compromised patients [28].

Although age was highlighted, in this study, as a significant predictor of adverse outcomes in patients who were infected and hospitalized (ward and ICU) due to COVID-19, impacting cognitive variables, it is important to consider age in conjunction with other factors, such as preexisting health conditions, for a comprehensive understanding of its influence on disease severity.

These data highlight the importance of a more in-depth age analysis as a significant factor in developing post-COVID cognitive problems. The relationship between age and cognitive impact may reveal nuances, providing insights for prevention and intervention strategies. Understanding how different age groups face and recover from these cognitive difficulties might guide personalized treatments and targeted support programs, considering the specific needs of each age group. Furthermore, a detailed investigation into this factor might aid in early risk identification and the development of more effective preventive measures for vulnerable populations.

Crivelli et al. [13], in a meta-analysis of 27 studies (resulting from 6,202 articles analyzed), cognitively assessed 2,049 individuals with a mean age of 56.05 years up to seven months after COVID-19 infection. Through a regression analysis, the authors found that an increase in age correlates with enhanced cognitive disfunction. It is possible that the presence of depressive symptoms in the moderate and severe groups may also contribute as a variable enhancing cognitive difficulty.

Our findings provide empirical evidence regarding cognitive effects post-COVID-19, particularly in individuals experiencing severe disease manifestations. Moreover, our study has demonstrated the presence of cognitive difficulties in individuals infected with mild and moderate symptoms, emphasizing the intricate nature of factors associated with COVID-19, as underscored in the existing literature [8, 10, 13, 15, 18, 19, 26, 29, 33, 49]. We also highlighted the need for a comprehensive, multidimensional approach in rehabilitation programs to address the enduring cognitive impacts of COVID-19.

### Study limitations

While our study presents an analysis encompassing three post-COVID-19 patient groups—mild, moderate, and severe—and provides an overarching understanding of cognitive performance, several limitations must be acknowledged. Primarily, the study’s cross-sectional design restricts the establishment of causal relationships. The absence of control for asymptomatic individuals within the mild symptoms group also represents a limitation. Assessing this subgroup, alongside the inclusion of an uninfected control group, could offer provide a more specific overview of the situation. Moreover, our study did not explore the impact of cognitive deficits on participants’ day-to-day functionality. Investigating these aspects could yield essential insights beneficial for bolstering cognitive rehabilitation programs.

## Conclusion

Our findings add additional empirical evidence toward understanding the long-term cognitive effects of COVID-19, particularly in individuals who experienced severe manifestations of the disease. Furthermore, we acknowledge the necessity for a multidimensional approach encompassing comprehensive investigation and assessment criteria as foundational elements for developing rehabilitation programs aimed at addressing the lasting cognitive repercussions of COVID-19.

## Data Availability

The datasets generated and/or analyzed during the current study are not publicly available due to data confidentiality but can be provided by the corresponding author upon reasonable request.
